# Analytical and Clinical Validation of a Serum microRNA RT-qPCR Assay for Detection of Acute Cellular Rejection in Liver Transplant Recipients

**DOI:** 10.3390/diagnostics16142152

**Published:** 2026-07-09

**Authors:** Yipeng Wang, Robert Huff, Haleigh Parker, Chang Han, Byung-In Lee, Shuguang Huang, Mackenzie Burke, Bao-Li Loza, Brendan J. Keating, Kim Olthoff, Abraham Shaked

**Affiliations:** 1 LuminoDx, San Diego, CA 92121, USA; 2 Penn Transplant Institute, Department of Surgery, University of Pennsylvania, Philadelphia, PA 19104, USA; 3 Department of Surgery, New York University, New York, NY 10016, USA

**Keywords:** liver transplantation, acute cellular rejection, microRNA, circulating biomarkers, liquid biopsy, molecular diagnostics, noninvasive monitoring

## Abstract

**Background:** Acute cellular rejection (ACR) remains a significant cause of graft dysfunction after liver transplantation and requires timely detection. Liver biopsy, the diagnostic gold standard for ACR, is invasive and unsuitable for frequent monitoring, while liver enzyme tests lack specificity for detecting ACR. Circulating microRNAs (miRNAs), including miR-122 and miR-885, have been previously identified as predictive biomarkers of ACR in liver transplant recipients. HepatoTrack™ is a serum-based miRNA RT-qPCR assay designed for noninvasive assessment of ACR using these biomarkers. This study evaluated the analytical performance and clinical validity of HepatoTrack™ for diagnosing ACR in liver transplant recipients. **Methods:** HepatoTrack™ uses 100 μL of serum and a one-step RT-qPCR workflow. It quantifies miR-122 and miR-885 normalized to miR-23a, with synthetic cel-miR-39 included as an exogenous control. Analytical validation assessed performance characteristics. Clinical performance was evaluated in a cohort of liver transplant recipients undergoing for-cause liver biopsy and used for model development and validation. **Results:** Analytical validation demonstrated robust assay performance. The HepatoTrack™ Prediction Score (HPS) algorithm was trained using 47 subjects from the training cohort based on a linear regression model incorporating longitudinal miRNA changes. In the independent testing cohort (*n* = 37), HPS achieved a sensitivity of 92.9%, specificity of 73.9%, positive predictive value (PPV) of 68.4%, and negative predictive value (NPV) of 94.4% for biopsy-confirmed ACR. HPS achieved an area under the curve (AUC) of 0.831 compared with 0.731 for ALT and 0.660 for AST. **Conclusions:** HepatoTrack™ support the analytical and clinical validity for detection of biopsy-confirmed acute cellular rejection in liver transplant recipients. The assay provides a noninvasive molecular test to aid in the diagnosis of acute cellular rejection and may complement existing post-transplant diagnostic evaluation.

## 1. Introduction

Liver transplantation remains the standard of care for patients with end-stage liver disease and selected hepatic malignancies. Despite advances in surgical technique and immunosuppressive therapy, acute cellular rejection (ACR) continues to occur and remains an important cause of graft dysfunction and long-term morbidity [1,2,3]. ACR results from immune-mediated injury to the transplanted liver and, if not recognized and treated in a timely manner, may lead to progressive graft damage and reduced graft survival [4]. Accurate and timely detection of ACR is therefore critical to guide immunosuppressive management and optimize post-transplant outcomes.

Liver biopsy remains the reference standard for diagnosing ACR. However, biopsy is invasive, carries procedural risks including bleeding and infection, and is subject to sampling variability and interobserver interpretation differences. In clinical practice, liver enzyme tests such as alanine aminotransferase (ALT) and aspartate aminotransferase (AST) are commonly used to monitor graft function, but these markers are nonspecific and may be elevated in a variety of non-rejection conditions, including ischemia–reperfusion injury, biliary complications, infection, or drug toxicity [5]. Consequently, abnormal liver enzyme levels often require biopsy for definitive diagnosis. These limitations highlight the need for noninvasive biomarkers capable of detecting immune-mediated graft injury with improved diagnostic specificity.

Circulating microRNAs (miRNAs) have emerged as promising biomarkers of organ-specific injury and immune activation [6,7]. These small non-coding RNA molecules regulate gene expression and are released into the circulation from injured tissues and activated immune cells, where they remain stable and can be reproducibly quantified using RT-qPCR–based methods. Prior studies have shown that circulating miRNA profiles can distinguish liver allograft rejection from non-rejection injury [8]. In particular, hsa-miR-122, a liver-enriched miRNA released during hepatocyte injury, and hsa-miR-885, which has been implicated in inflammatory and immune signaling pathways, were identified as key components of circulating miRNA signatures associated with acute cellular rejection (ACR) in liver transplant recipients [9]. Analyses of large transplant cohorts, including the ITN030ST and CTOT-03 studies [10,11], demonstrated that circulating miRNA signatures could distinguish rejection from non-rejection events and were detectable in serum prior to biopsy-confirmed rejection episodes [9]. These findings suggest that circulating miRNA measurements may provide clinically useful information for noninvasive detection of immune-mediated graft injury in liver transplant recipients.

HepatoTrack™ is a laboratory-developed test (LDT) that uses a one-step RT-qPCR platform to quantify serum miR-122 and miR-885, with normalization to endogenous miR-23a and inclusion of exogenous cel-miR-39 as a process control. The assay integrates longitudinal changes in miRNA levels into a composite HepatoTrack™ Prediction Score (HPS), designed to capture dynamic molecular signals associated with immune-mediated graft injury.

The objective of this study was to evaluate the analytical performance of HepatoTrack™ and its clinical validity for the diagnosis of acute cellular rejection in liver transplant recipients. We describe the analytical validation of the assay, evaluate its clinical performance for detection of biopsy-confirmed ACR, and compare its diagnostic performance with conventional liver enzyme measurements commonly used in post-transplant monitoring.

## 2. Materials and Methods

### 2.1. microRNA Target Panel

The assay targets four miRNAs: miR-122, miR-885, miR-23a, and cel-miR-39. miR-122 and miR-885 were selected based on prior studies demonstrating associations with liver-specific injury and immune activation, respectively, with additional internal verification [9]. miR-23a was used as an endogenous normalizer due to its stable expression in human serum [12,13]. Synthetic C. elegans cel-miR-39 was added to each sample prior to RNA extraction as an external process control.

### 2.2. Sample Collection, RNA Extraction, and RT-qPCR

Blood was collected in serum separator tubes and processed within 2 h. After centrifugation at 3000× *g* for 10 min, serum was aliquoted and stored at −80 °C.

For RNA extraction, 100 μL of serum was processed using the MagMAX™ mirVana™ Total RNA Isolation Kit on a KingFisher Duo Prime system (Thermo Fisher Scientific, Waltham, MA, USA). The final elution volume was 50 μL. cel-miR-39 was added prior to lysis.

miRNA quantification was performed using a one-step reverse transcription quantitative PCR (RT-qPCR) assay based on previously described SMOS-PCR technology [12]. The assay includes primer annealing, linker extension, and PCR amplification in a single-tube workflow. Reactions were run on a QuantStudio 7 Flex system (Thermo Fisher Scientific, Waltham, MA, USA). Each target was analyzed in triplicate on 384-well plates, and the median Ct value of the three technical replicates was used for downstream analysis. Samples with insufficient amplification or failing predefined quality-control criteria were repeated or excluded from analysis.

### 2.3. Quality Controls

Three types of quality control measures were incorporated to monitor assay performance throughout sample processing and RT-qPCR analysis.

RNA Extraction Control: Each extraction batch included an RNA isolation control (RIC; PBS containing cel-miR-39) to verify successful RNA extraction.

PCR Controls: Each RT-qPCR run included a Positive Template Control (PTC; synthetic miRNA mixture) to confirm successful amplification and a No Template Control (NTC; water) to monitor for contamination.

Sample Process Control: A fixed amount of synthetic cel-miR-39 was added to each serum sample prior to RNA extraction to monitor extraction efficiency and detect potential inhibitors affecting downstream RT-qPCR amplification.

Ct values from QC samples and cel-miR-39 process controls were evaluated against predefined acceptance criteria. Runs or samples failing QC criteria were repeated or excluded from analysis.

### 2.4. microRNA Target Panel Analytical Validation

Analytical validation of the HepatoTrack™ assay was performed in accordance with CLIA guidelines to evaluate LOB, LOD, quantification range (LLOQ and ULOQ), linearity and precision.

LOB was determined using a pool of non-target miRNA mimics (including hsa-miR-16, hsa-miR-21, hsa-miR-191, hsa-miR-192, hsa-miR-451, hsa-miR-483, hsa-miR-4790, and hsa-let-7a) tested at two concentration levels (24,000 and 240,000 copies per reaction), with 10 replicates at each level to characterize background signal and assay specificity.

LOD was established by testing serial dilutions of synthetic miRNA mimics at 1200, 2400, 12,000, and 24,000 copies per reaction, with 10 replicates per concentration.

The quantification range (LLOQ to ULOQ) was determined by combining data from the LOD study with additional higher concentration levels (240,000, 2,400,000, and 24,000,000 copies per reaction), tested in triplicate.

Assay precision was evaluated using 15 serum samples tested across three independent runs performed on different days, by different operators, and using different reagent lots. Each sample was tested in five replicates at three miRNA concentration levels. The low level consisted of unspiked serum from a normal subject, reflecting endogenous baseline expression. The medium and high levels were generated by spiking synthetic miRNA mimics into the same serum matrix at concentrations of 1.2 × 10^8^ copies for miR-122 and miR-23a and 1.2 × 10^7^ copies for miR-885 (medium), and 1.2 × 10^9^ copies for miR-122 and miR-23a and 1.2 × 10^8^ copies for miR-885 (high), to account for extraction losses and assay input fractions.

### 2.5. Clinical Cohort

Serum samples from liver transplant recipients were collected between 2012 and 2022 at the Penn Transplant Institute under Institutional Review Board (IRB)-approved protocols. Samples were stored at −80 °C in the University of Pennsylvania BioTIP biobank.

For each participant, two paired serum samples were obtained: one collected within three days prior to a for-cause liver biopsy (FCLB) and one collected at an earlier timepoint during routine follow-up. Histopathologic evaluation of the corresponding biopsy specimen was used as the reference standard to classify patients as ACR-positive or ACR-negative. Liver biopsy served as the reference standard for classification of acute cellular rejection (ACR). Histopathologic diagnoses were established according to routine clinical practice at the participating institution by board-certified pathologists, and patients were classified as ACR or non-ACR based on the final pathology diagnosis. Rejection severity was reported using the institutional pathology grading system (mild, moderate, or severe), when available. The HepatoTrack™ assay was performed retrospectively, and the assay results were not available at the time of clinical biopsy interpretation.

For model development and evaluation, the cohort was divided into a training set and an independent testing set. The training cohort included patients with a broader range of sampling intervals to enable model development using longitudinal miRNA dynamics. The independent testing cohort consisted of all patients meeting the predefined intended-use criteria (biopsy >180 days post-transplantation and longer longitudinal sampling intervals), thereby representing the intended clinical use population for HepatoTrack™.

### 2.6. Serum and Plasma Comparison Cohort

To evaluate potential sample type effects and assess the feasibility of using plasma with the HepatoTrack™ assay, a separate cohort of subjects with paired serum and plasma samples was analyzed. Blood samples were processed using standard protocols for serum and plasma separation, and miRNA extraction and RT-qPCR analysis were performed as described above. This analysis was conducted to assess concordance of miRNA measurements between sample types. Detailed cohort characteristics are provided in Supplemental File S1.

### 2.7. Blood Collection Tube Evaluation

To assess specimen stability during collection, storage, and transport, three blood collection tube types (EDTA, Streck Protein Plus BCT™, and Streck Cell-Free DNA BCT™) were evaluated under different storage durations and temperature conditions. Whole blood samples were assessed for miRNA stability using the HepatoTrack™ assay, and detailed study methods and results are provided in Supplemental File S2.

### 2.8. miRNA Normalization

Ct values for miR-122 and miR-885 were normalized to miR-23a using: ΔCt = Ct_target − Ct_miR-23a. Lower ΔCt values indicate higher expression. Cel-miR-39 was used for process monitoring only.

### 2.9. Statistical Analysis

Analyses were performed using R version 4.3.0. The LOB, LOD, LLOQ, ULOQ were determined in accordance with CLSI EP17-A2 guidelines, with detailed methods provided in Supplemental File S3. Model development was conducted using the caret and caretEnsemble packages. Several candidate prediction models, including classification and a regression tree (CART), logistic regression, and linear regression, were evaluated during model development.

A linear regression model incorporating longitudinal changes in normalized miR-122 and miR-885 values was used to generate the HepatoTrack™ Prediction Score (HPS), defined as:

$$HPS=\beta_{0}+\beta_{1}\times D_{miR122}+\beta_{2}\times D_{miR885}$$

where $D_{miR122}$ was defined as the difference between the normalized ΔCt value in the baseline sample and that in the test sample (baseline ΔCt − test ΔCt), and $D_{miR885}$ is similarly defined. Because lower ΔCt values indicate higher miRNA expression, a positive *D* value reflects an increase in miRNA expression relative to baseline. The regression coefficients were estimated from the training dataset, that higher HPS values reflect the pattern of longitudinal miRNA changes associated with biopsy-confirmed ACR.

The intercept (*β*_0_) was set to 0, allowing the HPS to primarily reflect changes in target miRNA expression, which could be interpreted as an approximate log_2_-transformed fold change. Under the assumption of 100% amplification efficiency, a 1-unit change in HPS corresponds to an approximate two-fold change in expression.

Receiver operating characteristic (ROC) curves and area under the curve (AUC) were calculated using the pROC package. Sensitivity, specificity, positive predictive value (PPV), negative predictive value (NPV), and balanced accuracy were derived from confusion matrices.

Normality of ΔCt distributions was assessed using the Shapiro–Wilk test. Because several variables deviated from normality, comparisons between ACR and non-ACR groups were performed using Wilcoxon rank-sum tests. *p* values < 0.05 were considered statistically significant.

## 3. Results

### 3.1. Analytical Performance: LOB, LOD, LLOQ, ULOQ, Linearity, and Precision

**Figure 1:**
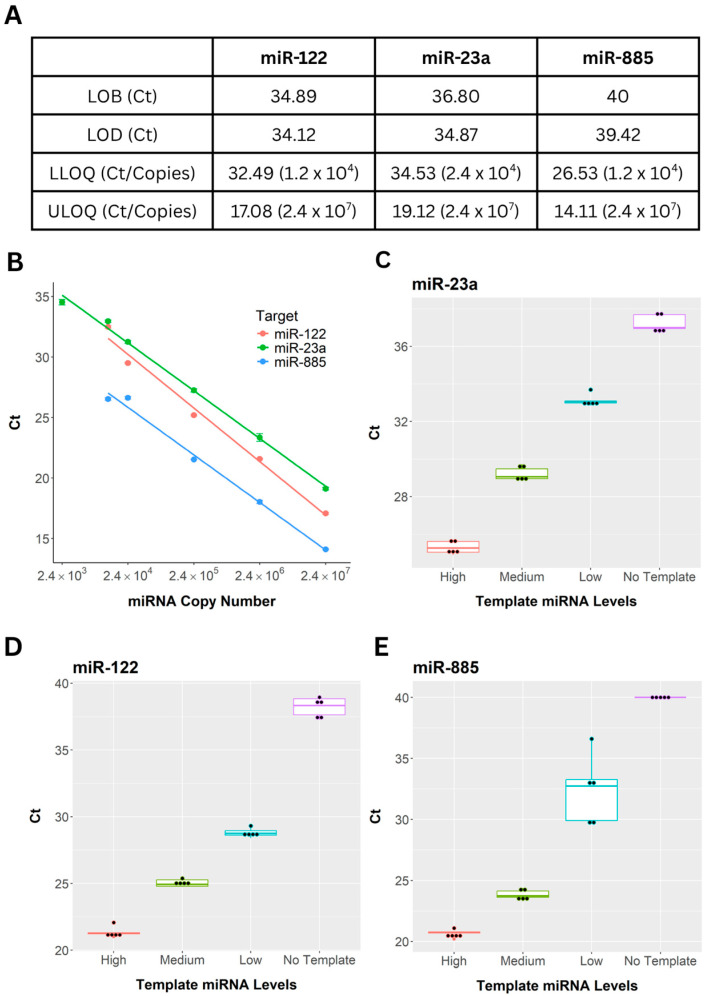
Analytical performance of the HepatoTrack™ assay. (**A**) Assay LOB, LOD, LLOQ and ULOQ using synthetic miRNA spike-in controls. (**B**) Linearity analysis for miR-122, miR-23a, and miR-885 across serial dilutions of synthetic miRNA templates spanning the assay’s dynamic range. Measured Ct values demonstrated a strong inverse linear relationship with input miRNA copy number, confirming assay linearity over multiple orders of magnitude. (**C**–**E**) Precision analysis across low, medium, and high miRNA concentration levels for miR-23a (**C**), miR-122 (**D**), and miR-885 (**E**) respectively. Boxplots show the distribution of Ct values for three target genes across replicate measurements, demonstrating low variability across runs, operators, and reagent lots.

Limits of blank (LoB), determined using non-target miRNA mimics, were 34.89 Ct for miR-122, 36.80 Ct for miR-23a, 40.00 Ct for miR-885, and 39.43 Ct for cel-miR-39, indicating minimal nonspecific amplification (Figure 1A).

Limits of detection (LoD), established using serial dilutions of synthetic miRNA templates, were 34.12 Ct for miR-122, 34.87 Ct for miR-23a, and 39.42 Ct for miR-885, demonstrating reliable detection of low-abundance targets (Figure 1A).

The assay exhibited a broad quantification range. The lower limit of quantification (LLOQ) was 12,000 copies per reaction for miR-122 and miR-885 and 2400 copies per reaction for miR-23a, corresponding to Ct values of 32.49, 26.53, and 34.53, respectively. The upper limit of quantification (ULOQ) extended to 2.4 × 10^7^ copies per reaction for all targets, supporting accurate quantification across multiple orders of magnitude (Figure 1A).

Assay linearity was evaluated by plotting input miRNA copy number against measured Ct values across the tested concentration range (Figure 1B). Linear regression analysis demonstrated excellent linearity for all three targets, with R^2^ values of 0.988 for miR-122, 0.994 for miR-23a, and 0.992 for miR-885.

Assay precision was evaluated across three runs performed on different days, by different operators, and using different reagent lots to assess repeatability and intermediate precision. Across all targets and concentration levels (low, medium, and high), the assay demonstrated low variability, with coefficients of variation (%CV) below 10% for miR-122 (3.41–5.33%), miR-23a (2.38–4.24%), and miR-885 (2.60–4.66%). The exogenous control cel-miR-39 showed slightly higher variability (%CV = 8.92%) but remained within acceptable limits (Figure 1C–E).

### 3.2. Clinical Cohort Characteristics

Of the 84 patients, 47 were assigned to the training cohort (30 non-ACR and 17 ACR), and 37 were assigned to the independent testing cohort (23 non-ACR and 14 ACR). The testing cohort consisted of patients with biopsy samples obtained more than 180 days post-transplantation and with longer intervals between paired serum samples (>60 days), representing a more clinically stable population consistent with the intended clinical application of the HepatoTrack™ assay.

The mean age of the cohort was 55.1 years, and 64% were male. There were no significant differences in age, sex, or time post-transplantation between the training and testing cohorts. Detailed demographic and clinical characteristics are summarized in Table 1. The combined non-ACR cohort comprised patients with a broad spectrum of clinically relevant post-transplant liver diseases, including steatosis/steatohepatitis, chronic or recurrent hepatitis, biliary complications, portal venopathy and other vascular abnormalities, cholestasis/cholangitis, nodular regenerative hyperplasia, and other inflammatory liver disorders (Supplemental File S4).

### 3.3. Differential miRNA Expression

**Figure 2:**
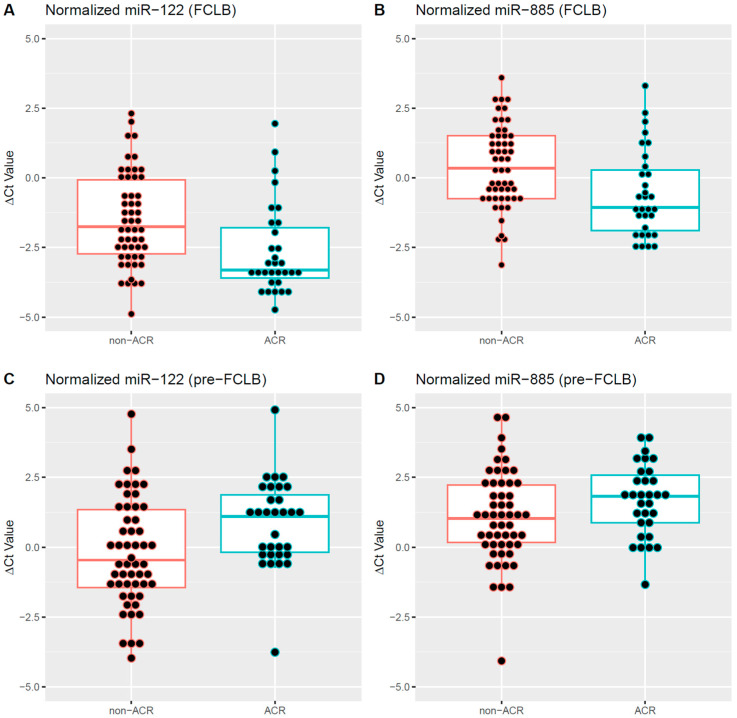
Boxplots comparing normalized expression of miR-122 and miR-885 between ACR and non-ACR groups at two sampling timepoints (FCLB and pre-FCLB). miR-122 is shown in panels (**A**) (FCLB) and (**C**) (pre-FCLB), and miR-885 is shown in panels (**B**) (FCLB) and (**D**) (pre-FCLB). The x axis denotes rejection status, and the y-axis represents normalized ΔCt values. Individual data points are overlaid on each boxplot.

At the FCLB timepoint, normalized ΔCt values for miR-122 and miR-885 were significantly lower in ACR patients compared with non-ACR patients (miR-122: *p* = 0.01; miR-885: *p* = 0.03) (Figure 2A,B), consistent with increased expression of both miR-122 and miR-885 in the setting of ACR.

At the Pre-FCLB timepoint, miR-122 remained significantly different between groups (*p* = 0.01), whereas miR-885 did not reach statistical significance (*p* = 0.23) (Figure 2C,D). However, both miRNAs exhibited an opposite directional trend compared with that observed at the FCLB timepoint.

### 3.4. Prediction Model Development and Performance Evaluation

The prediction model was developed using the training set (*n* = 47; 30 non-ACR and 17 ACR).

Multiple modeling approaches implemented through the caretEnsemble package were evaluated for clinical performance. Among these, CART, logistic regression, and linear regression demonstrated comparable performance. Because the linear regression model achieved performance similar to that of CART and logistic regression while yielding a simpler formula and a more readily interpretable HPS, it was selected as the final model. Detailed comparisons of the candidate prediction models, including their performance and the rationale for selecting the final linear regression model, are provided in Supplemental File S5.

Two modeling strategies were assessed: (1) using FCLB sample measurements alone, and (2) incorporating longitudinal changes between the FCLB sample and a pre-FCLB/baseline sample. The latter approach demonstrated superior performance and was selected for the final model.

The final linear regression model incorporates longitudinal changes in normalized miR-122 and miR-885 values to generate the HPS, with coefficients *β*_1_ (0.8) and *β*_2_ (0.2) derived from the training dataset. Because lower ΔCt values indicate higher miRNA expression, positive Change values correspond to increased miRNA expression relative to baseline. The positive coefficients for both miRNAs therefore assign higher HPS values to patients exhibiting greater longitudinal increases in miRNA expression, and a higher HPS corresponds to a greater likelihood of ACR.

**Figure 3:**
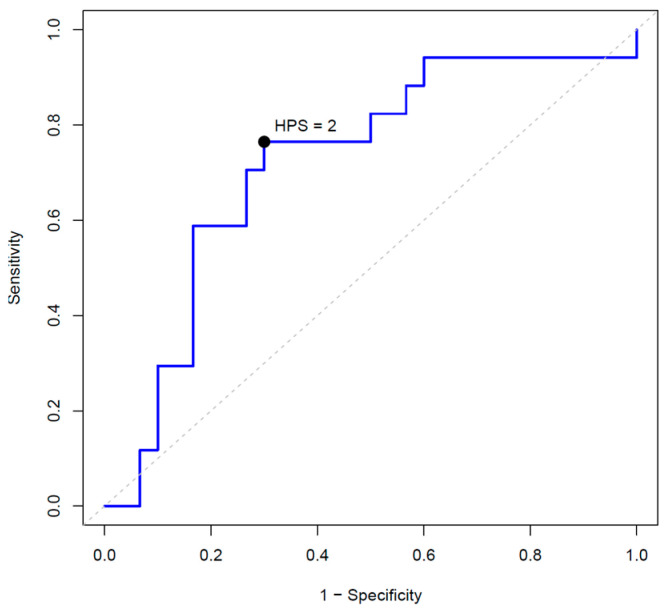
ROC curve for HPS cutoff selection in the training cohort. The ROC analysis yielded an AUC of 0.720 (95% CI: 0.561–0.878). A cutoff of 2 was selected to balance sensitivity (76%) and specificity (70%).

An HPS cutoff value of 2 was selected based on ROC curve analysis in the training cohort, balancing sensitivity (76%) and specificity (70%). The ROC analysis yielded an AUC of 0.720 (95% CI: 0.561–0.878, DeLong method). The corresponding positive predictive value (PPV) and negative predictive value (NPV) were 59% and 84%, respectively (Figure 3).

Model performance was evaluated in the independent testing subset (*n* = 37; 23 non-ACR and 14 ACR). Using the predefined cutoff, the model achieved a sensitivity of 92.8%, specificity of 73.9%, positive predictive value (PPV) of 68.4%, negative predictive value (NPV) of 94.4%.

The confusion matrices and corresponding diagnostic performance metrics for the linear regression model in the training and independent testing cohorts are summarized in Table 2.

### 3.5. Comparison of HPS with ALT and AST

**Figure 4:**
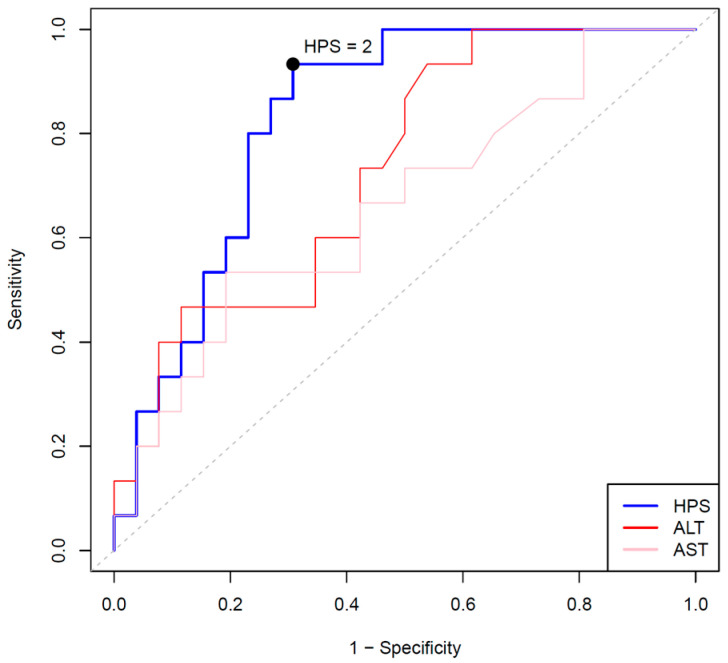
ROC analysis comparing the diagnostic performance of HPS, ALT, and AST in the independent test cohort. The AUC for HPS was 0.831 (95% CI: 0.707–0.955), compared with 0.731 (95% CI: 0.575–0.887) for ALT and 0.660 (95% CI: 0.482–0.839) for AST.

ROC curve analysis was performed to compare the discriminatory performance of HPS with standard-of-care liver enzyme measurements using the combined validation cohort. The AUC for HPS was 0.831 (95% CI: 0.707–0.955, DeLong method), compared with 0.731 (95% CI: 0.575–0.887, DeLong method) for alanine aminotransferase (ALT) and 0.660 (95% CI: 0.482–0.839, DeLong method) for aspartate aminotransferase (AST). Although the AUC for HPS was numerically higher than that of ALT and AST, the difference did not reach statistical significance (*p* = 0.286 and 0.096 respectively) due to limited sample size. ROC curves for HPS, ALT, and AST are shown in Figure 4.

### 3.6. Comparison of Serum and Plasma Samples

To evaluate potential matrix effects, 42 paired serum and plasma samples from a separate cohort were analyzed to assess concordance of miRNA measurements. Correlation analysis demonstrated a high degree of agreement between sample types for all three targets, with no evidence of systematic bias across the measured range (Supplemental File S1 Figure S1). The correlation coefficients were 0.93 for miR-122, 0.97 for miR-885, and 0.75 for miR-23a. The observed variability between matrices was minimal relative to the overall dynamic range of the assay.

HPS values were further evaluated in a subset of 10 patients with longitudinal blood draws. HPS values derived from serum and plasma samples were strongly correlated (r = 0.82), and all patients were classified consistently (8 ACR and 2 non-ACR) across both sample types (Supplemental File S1 Figure S2).

These results indicate that miRNA quantification using the HepatoTrack™ assay is consistent across serum and plasma matrices, supporting the robustness of the assay to sample type.

### 3.7. Specimen Stability

Specimen stability was evaluated to support clinical implementation of the HepatoTrack™ assay. Among the blood collection tubes evaluated, Streck Protein Plus BCT™ demonstrated superior preservation of circulating miRNA profiles during whole-blood storage compared with standard EDTA and Streck Cell-Free DNA BCT™ tubes. Ambient-temperature stability studies showed that longitudinal HPS values remained within 1 unit of baseline for up to 2 days, whereas greater variability was observed with longer storage durations. Additional simulated shipping studies demonstrated relatively stable HPS values following storage at temperatures ranging from 4 °C to 32 °C for up to 48 h. Based on these findings, plasma isolation within 2 days of blood collection is recommended for optimal assay performance. Detailed methods and results are provided in Supplemental File S2.

## 4. Discussion

The HepatoTrack™ assay demonstrated clinical validity for the detection of biopsy-confirmed acute cellular rejection (ACR) in liver transplant recipients undergoing diagnostic evaluation for suspected allograft dysfunction within this retrospective cohort. By quantifying circulating microRNA biomarkers associated with hepatocellular injury (miR-122) and immune activation (miR-885), the assay provides molecular information intended to complement existing diagnostic approaches and address limitations of current monitoring strategies.

Recent studies have continued to expand the potential applications of circulating miRNAs as biomarkers across a broad spectrum of liver diseases. For example, a recent study evaluating fecal microbiota transplantation in patients with alcohol-related cirrhosis demonstrated dynamic changes in circulating miRNA expression associated with clinical and microbiome responses, supporting the concept that circulating miRNAs reflect biologically meaningful changes in liver disease and immune regulation. Although that study addressed a different clinical setting than liver transplantation and acute cellular rejection, it reinforces the broader potential of circulating miRNAs as dynamic biomarkers and provides additional context for the development of miRNA-based diagnostic assays in hepatology [14].

miR-23a has been shown to maintain stable expression in circulating biofluids and is commonly incorporated into normalization strategies for serum miRNA assays. It has been used both as an individual reference control and in combination with other markers, including its established role as the reference miRNA in hemolysis assessment frameworks utilizing miR-451a as a hemolysis-sensitive marker, as well as in studies of circulating miRNA assay standardization [15,16,17,18]. To further evaluate its suitability as an endogenous normalizer in liver transplant recipients, we analyzed an independent dataset comprising more than 300 serum samples collected from liver transplant recipients during assay development (Supplemental File S6). Among 20 candidate endogenous control miRNAs evaluated, miR-23a demonstrated low expression variability, with a coefficient of variation below 4% and a Ct range of less than 10 cycles across samples. Comparison between ACR and non-ACR groups showed only a small difference in mean Ct values (ΔCt = −0.4), among the smallest observed across all candidate endogenous controls, supporting its relative stability across clinical states. In this assay, miR-23a serves as the primary normalizer, while longitudinal changes in miRNA expression are incorporated into the prediction model to mitigate inter-individual variability and capture dynamic molecular signals associated with evolving graft status.

HPS primarily reflects changes in target miRNA expression, which can be interpreted as a log_2_-transformed fold change. Under the assumption of 100% amplification efficiency, a 1-unit change in HPS corresponds to an approximate two-fold change in expression.

The training and independent testing cohorts were intentionally defined to reflect distinct clinical and temporal characteristics to support both model development and independent performance evaluation. The training cohort included a broader range of patients with shorter intervals between longitudinal samples, enabling the model to learn dynamic changes in miRNA expression. In contrast, the independent testing cohort consisted of patients with biopsy samples obtained more than 180 days post-transplantation and longer intervals between paired serum samples because this population more closely reflects the intended clinical application of the HepatoTrack™ assay, namely evaluation of established liver transplant recipients undergoing assessment for suspected allograft dysfunction. Consequently, the observed diagnostic performance should be interpreted within the context of this intended-use population.

Analytically, the assay demonstrated robust performance characteristics, including linearity across the reportable range, low variability, and well-defined limits of detection and quantification. Additional studies evaluating specimen handling and transport further supported the robustness of the assay under anticipated clinical workflow conditions. These features, together with the streamlined one-step RT-qPCR workflow and integrated quality controls, support reliable implementation as a laboratory-developed test (LDT) in a clinical laboratory setting. The simplified workflow also supports scalability and potential automation, which may facilitate broader clinical adoption.

In the diagnostic evaluation setting, the HPS demonstrated high sensitivity and negative predictive value, supporting its potential role as a noninvasive rule-out tool to reduce unnecessary biopsies in patients with suspected graft dysfunction. Although the independent test cohort demonstrated higher sensitivity than the training cohort, this difference should be interpreted cautiously because both cohorts were modest in size and the corresponding confidence intervals overlap, suggesting that the observed difference may reflect sampling variability rather than a true improvement in performance. Notably, the use of longitudinal miRNA changes enables detection of dynamic biological signals associated with immune-mediated injury, which may not be captured by static measurements alone.

ROC analysis demonstrated that HPS achieved higher discriminatory performance than ALT and AST in the validation cohort. These findings suggest that the miRNA-based composite score provides diagnostic performance comparable to conventional liver enzyme testing in this study while reflecting immune-mediated biological processes not fully captured by transaminases alone. As such, this approach may provide complementary information to existing laboratory tests and improve clinical decision-making.

Several limitations should be acknowledged. The study was retrospective and based on samples from a single biobank, which may limit generalizability. The sample size was modest, and variability in sampling intervals and timing relative to clinical events may introduce heterogeneity. In addition, circulating miR-122 may be influenced by causes of liver injury other than acute cellular rejection. Although the non-ACR cohort included a broad spectrum of clinically relevant post-transplant liver diseases—including steatosis/steatohepatitis, chronic or recurrent hepatitis, biliary complications, vascular abnormalities, cholangitis, and other inflammatory liver disorders—larger prospective studies with detailed clinical phenotyping and multivariable analyses will be important to further define the specificity and incremental clinical value of the HepatoTrack™ assay across diverse causes of allograft dysfunction. Furthermore, liver biopsy served as the reference standard and was interpreted according to routine clinical pathology practice at a single institution. Standardized Banff scoring, formal assessment of interobserver variability, and systematic evaluation of borderline or indeterminate rejection were not available in this retrospective dataset. As with liver biopsy in general, sampling variability and interobserver interpretation may have influenced the reference standard classification and, consequently, the apparent performance of the assay. Prospective, multicenter studies conducted by independent investigators and incorporating standardized histopathologic review will be important to further define the performance characteristics, generalizability, and clinical utility of the HepatoTrack™ assay in routine practice.

In addition, comparable results were observed between serum and plasma samples in a separate cohort, supporting the robustness of the assay across sample matrices. These findings are consistent with prior reports demonstrating minimal differences between serum and plasma in circulating miRNA analysis [19]. In clinical practice, however, a consistent sample type should be used for each patient to ensure valid longitudinal comparisons.

## 5. Conclusions

These findings support the analytical and clinical validity of HepatoTrack™ as a noninvasive molecular tool for detection of biopsy-confirmed acute cellular rejection in liver transplant recipients. Prospective multicenter studies will be valuable to further evaluate assay performance across broader transplant populations and clinical practice settings.

## Figures and Tables

**Table 1:** Clinical characteristics of the study cohort.

	All Patients	Training Set	Test Set
**Patient Number**	84	47	37
**Age at Transplantation (Year)**	23–72, average 55.1	35–72, average 57.0	23–69, average 52.9
**Gender**	M (54), F (30)	M (32), F (15)	M (22), F (15)
**Race**	White (65), Black or African American (12), HLW-Hispanic Latino/White (2), Some Other Race (4), White, Native Hawaiian or Other Pacific Islander (1)	White (38), Black or African American (6), Some Other Race (2), HLW-Hispanic Latino/White (1)	White (27), Black or African American (6), HLW-Hispanic Latino/White (1), Some Other Race (2), White, Native Hawaiian or Other Pacific Islander (1)
**Ethnicity**	Not Hispanic or Latino (82), Hispanic Latino (2)	Not Hispanic or Latino (46), Hispanic Latino (1)	Not Hispanic or Latino (36), Hispanic Latino (1)
**Days Between Liver Biopsy and Transplantation Date**	36–2177, average 402	36–1670, average 183	181–2177, average 681
**Days Between Longitudinal Blood Draws**	1–716, average 150	1–91, average 37	64–716, average 293
**ACR Status**	No-ACR (53), ACR (31)	No-ACR (30), ACR (17)	No-ACR (23), ACR (14)

**Table 2:** Confusion Matrix and Performance Statistics for Linear Regression Model Prediction on the Training Cohort.

	Training Set	Test Set
**Sample Size (*n*)**	47	37
**True Positive (ACR)**	13	13
**False Positive**	9	6
**False Negative**	4	1
**True Negative (non-ACR)**	21	17
**Accuracy (95% CI)**	72.3% (57.4–84.4%)	81.1% (64.8–92.0%)
**Balanced Accuracy**	73.2%	83.4%
**Sensitivity**	76.5% (50.1–93.2%)	92.9% (66.1–99.8%)
**Specificity**	70.0% (50.6–85.3%)	73.9% (51.6–89.8%)
**Pos Pred Value**	59.1% (36.4–79.3%)	68.4% (43.4–87.4%)
**Neg Pred Value**	84.0% (63.9–95.5%)	94.4% (72.7–99.9%)

## Data Availability

The data presented in this study are available from the corresponding author upon reasonable request. The data are not publicly available due to patient privacy and institutional ethical restrictions.

## Supplementary Materials

The following supporting information can be downloaded at: https://www.mdpi.com/article/10.3390/diagnostics16142152/s1, File S1: Evaluation of Serum and Plasma Specimen Equivalency for the HepatoTrack™ Assay; File S2: Blood Collection Tube Evaluation; File S3: Determination of LOB, LOD, LLOQ, and ULOQ for the HepatoTrack™ Assay According to CLSI EP17-A2 Guidelines; File S4: Summary of Histopathologic Diagnoses in Non-ACR Patients; File S5: Evaluation and Selection of Prediction Models for HepatoTrack™ Prediction Score Development; File S6: Evaluation of Circulating miRNA Endogenous Controls in Liver Transplant Recipient Serum Samples.

## Abbreviations

The following abbreviations are used in this manuscript:

miRNA	microRNA
ACR	Acute Cellular Rejection
CART	Classification and a Regression Tree
CO	Confidence Interval
HPS	HepatoTrack™ Prediction Score
LOB	Limit of Blank
LOD	Limit of Detection
LLOQ	Lower Limit of Quantitation
ULOQ	Upper Limit of Quantitation
LDT	Laboratory Developed Test
NPV	Negative Predictive Value
PPV	Positive Predictive Value
FCLB	For-cause Liver Biopsy
