# Supplementary material for: Analytical and Clinical Validation of a Serum microRNA RT-qPCR Assay for Detection of Acute Cellular Rejection in Liver Transplant Recipients
Source: Diagnostics (Basel). 2026 Jul 9;16(14):2152. doi: 10.3390/diagnostics16142152 (PMC13409392; doi:10.3390/diagnostics16142152)
Supplement: Supplementary file 1 [file diagnostics-16-02152-s001.zip › HepatoTrack Analytical Clinical Validation Supplemental Material S1.pdf]

Supplemental File S1

Evaluation of Serum and Plasma Specimen Equivalency for the HepatoTrack™ Assay

Cohort Summary

A total of 84 samples, comprising 42 paired serum and plasma specimens, were collected from post-transplant patients at the University of Pennsylvania. Among these, 20 paired serum and plasma samples were obtained at two independent time points from 10 study subjects (baseline and follow-up), enabling longitudinal comparison of target miRNA expression, HepatoTrack™ Prediction Score (HPS), and its predefined risk threshold between serum and plasma. All samples were processed and analyzed with HepatoTrack™ assay.

Plasma vs. Serum Comparison at Individual Target Level

The correlation coefficients for the paired comparisons were 0.93 for miR-122, 0.97 for miR-885 and 0.75 for miR-23a. **Supplemental File S1 Figure S1** shows the corresponding scatter plots. The slightly lower correlation for miR-23a reflects its narrower dynamic range and its more stable, housekeeping-like expression pattern.

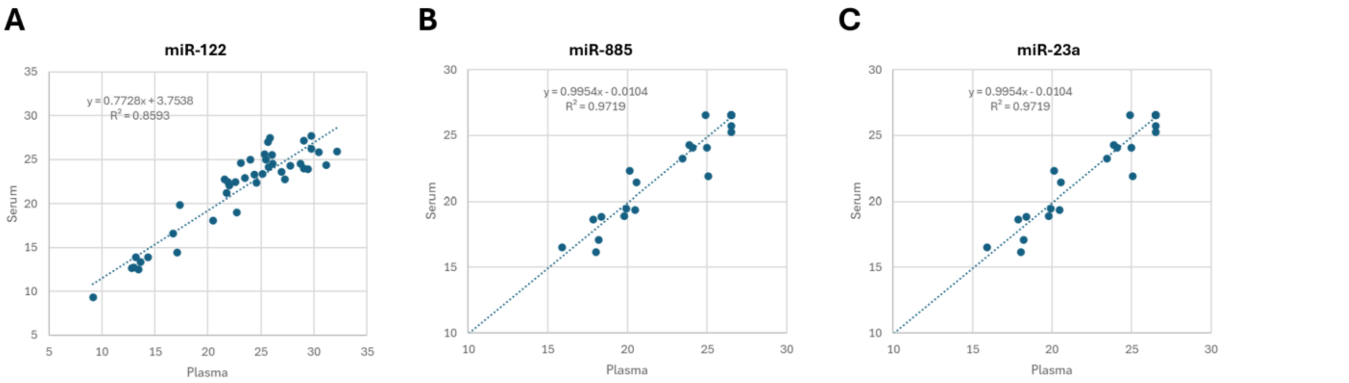

Supplemental File S1 Figure S1: Comparison of miRNA measurements in 42 paired plasma and serum samples: (A) miR-122, (B) miR-885, and (C) miR-23a.

The average differences between plasma and serum were –1.50 (range: –6.83 to 2.48) for miR-122, –0.12 (range: –3.18 to 2.20) for miR-885, and –1.83 (range: –8.01 to 2.31) for miR-23a. On average, miRNA levels in plasma samples were 1–2 Ct higher (i.e., lower abundance) than in serum samples, indicating sample type specific differences at the individual analyte level in this cohort. These differences may stem from factors such as sample processing time, preservative stability, other pre-analytical variables, and variations in RNA isolation efficiency.

Assessment of Plasma-Serum Agreement at HPS Level by Bland-Altman Analysis

Agreement between serum- and plasma-derived HepatoTrack Prediction Scores (HPS) was further evaluated using Bland–Altman analysis. The mean bias (plasma − serum) was −0.02 HPS units, indicating negligible systematic difference between the two specimen types. The 95% limits of agreement ranged from −1.73 to 1.69 HPS units, as shown in **Supplemental File S1 Figure S2**. Linear regression analysis of the Bland–Altman differences demonstrated no evidence of proportional bias across the HPS measurement range (slope = 0.064, $p = 0.783$).

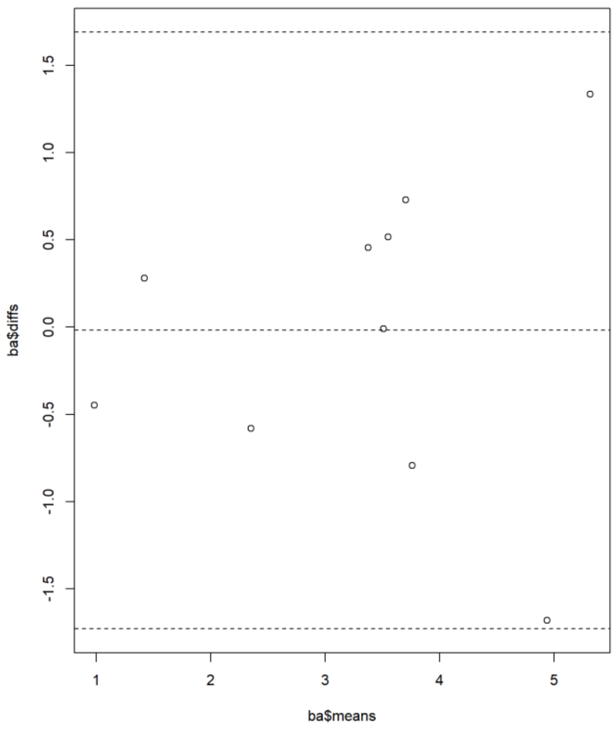

Supplemental File S1 Figure S2. Bland–Altman analysis of HPS derived from paired plasma and serum samples. The solid horizontal line indicates the mean bias (−0.02 HPS units), and the dashed lines represent the 95% limits of agreement (−1.73 to 1.69 HPS units).

Serum- and plasma-derived HPS values were strongly correlated (Pearson r = 0.82). Consistent with the Bland–Altman analysis, application of the predefined HPS cutoff yielded 100% concordance in rejection risk classification between serum and plasma samples for all paired longitudinal specimens (**Supplemental File S1 Figure S3)**.

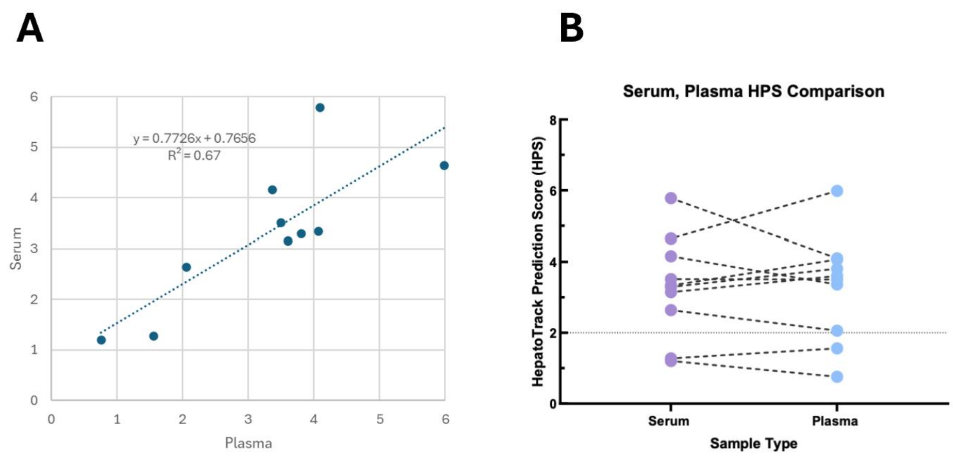

Supplemental File S1 Figure 3: Comparison of HPS and rejection classification between paired plasma and serum samples from the longitudinal cohort. (A) Scatter plot of plasma versus serum HPS values for 10 paired specimens collected at baseline and follow-up. (B) Concordance of rejection classification between plasma and serum using the predefined HPS cutoff of 2.

Conclusion

Overall, these findings demonstrate good agreement between serum- and plasma-derived HPS measurements and support the use of either specimen type for the HepatoTrack™ assay. Although individual miRNA Ct values differed modestly between specimen types, these differences had minimal impact on the composite HPS and did not alter clinical classification in the paired longitudinal cohort. Nevertheless, a consistent specimen type should be maintained for longitudinal patient monitoring.
