# Supplementary material for: Analytical and Clinical Validation of a Serum microRNA RT-qPCR Assay for Detection of Acute Cellular Rejection in Liver Transplant Recipients
Source: Diagnostics (Basel). 2026 Jul 9;16(14):2152. doi: 10.3390/diagnostics16142152 (PMC13409392; doi:10.3390/diagnostics16142152)
Supplement: Supplementary file 1 [file diagnostics-16-02152-s001.zip › HepatoTrack Analytical Clinical Validation Supplemental Material S2.pdf]

Supplemental File S2

Blood Collection Tube Evaluation

To support clinical implementation of the HepatoTrack™ assay, three whole-blood collection tube types were evaluated for specimen stability during storage and transport: standard EDTA (purple-top) tubes, Streck Cell-Free DNA BCT™ (formerly Streck Nucleic Acid™), and Streck Protein Plus BCT™ tubes. Plasma was isolated according to either the standard plasma preparation protocol or the manufacturer's recommended protocol for each collection tube, and all specimens were subsequently analyzed using the HepatoTrack™ assay.

Preliminary Evaluation of Blood Collection Tubes

An initial comparison of the three blood collection tube types was performed to identify the most suitable specimen collection method for circulating miRNA analysis. EDTA tubes exhibited suboptimal miRNA stability after 24 hours of ambient-temperature storage, with raw miR-23a Ct values increasing by more than 3 Ct compared with Day 0 measurements. Streck Cell-Free DNA BCT™ tubes also demonstrated reduced stability during ambient-temperature storage, with visible hemolysis observed by Day 3. In contrast, Streck Protein Plus BCT™ tubes demonstrated superior preservation of circulating miRNA profiles and were therefore selected for subsequent specimen stability studies.

Ambient-Temperature Stability of Streck Protein Plus BCT™

To evaluate the suitability of Streck Protein Plus BCT™ tubes for circulating miRNA analysis, whole-blood samples from six individuals were collected into Streck Protein Plus BCT™ tubes and stored at ambient temperature for up to seven days prior to plasma separation. Specimen stability was assessed using the HepatoTrack™ Prediction Score (HPS). For this analysis, the Day 0 sample from each subject was designated as the baseline specimen, and samples collected at subsequent time points (Days 1, 2, 3, 5, and 7) were analyzed as follow-up specimens to calculate longitudinal HPS values.

Supplemental File S2 Table S1. HPS values for plasma prepared from Streck Protein Plus BCT™ tubes following ambient-temperature storage.

	**Subject A**	**Subject B**	**Subject C**	**Subject D**	**Subject E**	**Subject F**
**Day 0**	0	0	0	0	0	0

**Day 1**	-0.103	-0.366	0.366	ND	ND	ND
**Day 2**	-0.879	-0.629	0.629	-0.522	-0.286	ND
**Day 3**	-1.224	0.594	-0.594	ND	ND	-1.917
**Day 5**	ND	ND	ND	ND	ND	-2.888
**Day 7**	ND	ND	ND	ND	ND	-3.055

ND: No data.

HPS values at each time point were compared with the corresponding Day 0 baseline. Differences remained within 1 HPS unit through Day 2. Beginning on Day 3, greater variability was observed, and HPS values progressively diverged from baseline with increasing storage duration (Supplemental File S2 Figure S1). Although Streck Protein Plus BCT™ tubes are intended by the manufacturer for preservation of plasma proteins for up to five days under ambient conditions, these data indicate that optimal preservation of circulating miRNA profiles for the HepatoTrack™ assay is achieved when plasma is isolated within two days after blood collection.

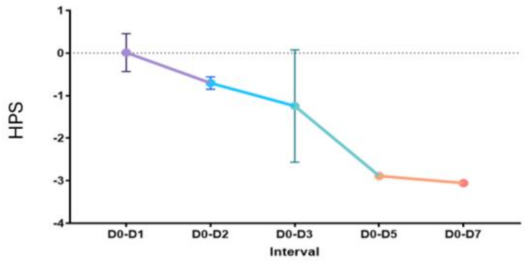

Supplemental File S2 Figure S1. Ambient-temperature stability of circulating miRNA assessed using the HepatoTrack™ Prediction Score (HPS).

Simulated Shipping Temperature Stability

To evaluate specimen stability under anticipated shipping conditions, whole-blood samples from four individuals were collected into Streck Protein Plus BCT™ tubes and stored at 4°C, 20°C (ambient temperature), or 32°C for either 24 or 48 hours prior to plasma separation. Specimen stability was assessed using the HPS. For each subject, the sample stored at 20°C served as the reference specimen, and HPS differences were calculated for the corresponding samples stored at 4°C or 32°C under the same storage duration.

Supplemental File S2 Table S2. HPS differences following storage under simulated shipping temperature conditions.

	24 Hours			48 Hours		
	Subject A	Subject B	**Average**	Subject C	Subject D	**Average**
**4°C vs. 20°C**	-1.761	-0.81	-1.286	-1.155	-1.794	-1.475
**32°C vs. 20°C**	-0.696	-0.365	-0.531	-1.656	-1.946	-1.801

Compared with the ambient-temperature reference (20°C), HPS differences after 24 hours remained below 2 units under both refrigerated (4°C) and elevated-temperature (32°C) storage conditions. Larger HPS differences were observed after 48 hours, with average changes exceeding 1 HPS unit under both temperature conditions. Overall, these findings suggest that the HepatoTrack™ assay is relatively robust to storage temperatures ranging from 4°C to 32°C for up to 48 hours, although prolonged storage is associated with increased variability in HPS.

These findings support the use of Streck Protein Plus BCT™ tubes for short-term specimen transport under anticipated shipping temperatures, while reinforcing the recommendation that plasma be isolated within 48 hours of blood collection.

Summary

These studies demonstrate that Streck Protein Plus BCT™ tubes provide improved preservation of circulating miRNA profiles compared with EDTA and Streck Cell-Free DNA BCT™ tubes under ambient-temperature storage conditions. For the HepatoTrack™ assay, plasma separation within two days after blood collection is recommended to maintain optimal miRNA stability. The assay also demonstrated relative stability following storage at temperatures ranging from 4°C to 32°C for up to 48 hours, supporting the feasibility of specimen transport under anticipated clinical shipping conditions. In the intended clinical workflow, whole-blood specimens are shipped in insulated packaging designed to minimize temperature fluctuations during transport, further supporting maintenance of specimen integrity. Additional studies using larger sample sets and real-world shipping conditions will further strengthen these observations.
