# Supplementary material for: Analytical and Clinical Validation of a Serum microRNA RT-qPCR Assay for Detection of Acute Cellular Rejection in Liver Transplant Recipients
Source: Diagnostics (Basel). 2026 Jul 9;16(14):2152. doi: 10.3390/diagnostics16142152 (PMC13409392; doi:10.3390/diagnostics16142152)
Supplement: Supplementary file 1 [file diagnostics-16-02152-s001.zip › HepatoTrack Analytical Clinical Validation Supplemental Material S3.pdf]

Supplemental File S3

Determination of LOB, LOD, LLOQ, and ULOQ for the HepatoTrack™ Assay According to CLSI EP17-A2 Guidelines

LOB:

To calculate the LOB, the normality of the data was evaluated by Shapiro-Wilk test (13). LOB was then determined using a parametric approach and the formulars are shown below.

$$LoB = M_B - C_p SD_B$$

$$C_p = \frac{1.645}{1 - (\frac{1}{4(B-K)})}$$

where, C_p is a multiplier to give 95th percentile of a normal distribution, B = total number of blank sample results in the dataset, K = number of blank samples.

LOD:

The LOD was defined by the lowest concentration that could be reliably distinguished from the blank. The LOD was determined using a standard approach by fitting a precision profile model, in which the standard deviations of repeated measurements are plotted on the y-axis against the corresponding mean measurand concentrations on the x-axis. The precision profile model and LOD are defined as follows:

$$SD_{WL} = B_1 + B_2 X + B_3 X^2$$

$$LoD = LoB + C_p SD_{WL}$$

$$C_p = \frac{1.645}{1 - (\frac{1}{4(N_{TOT}-K)})}$$

where SD_{WL} represents within-laboratory precision, X is the measurand concentration, and B_1, B_2, and B_3 are parameters to be estimated in the model fit process. K = number of precision studies.

LLOQ and ULOQ:

The quantification ranges were established using spike-in samples prepared by adding known concentrations of synthetic target RNAs into the blank matrix. The LLOQ was defined as the lowest concentration that satisfied the %CV < 15%. If the calculated LOQ was lower than the LOD, the lowest LOD concentration that satisfied the %CV < 15% was selected as the final LOQ. The ULOQ was defined as the highest concentration within the assay's linear range that met precision acceptance criteria. The LLOQ and ULOQ together established the assay's measurement range.
