# Supplementary material for: Analytical and Clinical Validation of a Serum microRNA RT-qPCR Assay for Detection of Acute Cellular Rejection in Liver Transplant Recipients
Source: Diagnostics (Basel). 2026 Jul 9;16(14):2152. doi: 10.3390/diagnostics16142152 (PMC13409392; doi:10.3390/diagnostics16142152)
Supplement: Supplementary file 1 [file diagnostics-16-02152-s001.zip › HepatoTrack Analytical Clinical Validation Supplemental Material S4.pdf]

Supplemental File S4

Summary of Histopathologic Diagnoses in Non-ACR Patients

The non-ACR cohort comprised patients undergoing clinically indicated liver biopsy for suspected graft dysfunction. Histopathologic findings represented a broad spectrum of post-transplant liver diseases and are summarized below.

Supplemental File S4 Table S1. Summary of Histopathologic Diagnoses in Non-ACR Patients

Principal histopathologic finding *	Training cohort (n = 30)	Test cohort (n = 23)	Total (n = 53)
**Steatosis / steatohepatitis**	7	6	13
**Chronic or recurrent hepatitis**	7	5	12
**Biliary complications †**	5	5	10
**Portal venopathy / vascular abnormalities ‡**	5	5	10
**Cholestasis / cholangitis**	3	3	6
**Nodular regenerative hyperplasia**	3	2	5
**Other inflammatory or immune-mediated conditions ¶**	3	4	7

* Categories are not mutually exclusive; individual patients may have more than one histopathologic diagnosis. Mild portal, periportal, perisinusoidal, pericellular, or bridging fibrosis was commonly observed in association with the diagnoses listed above.

† Includes ischemic cholangiopathy, biliary obstruction, bile duct injury, biloma, bile duct anastomotic stricture, and pericholangitis.

‡ Includes portal tract venopathy, venous outflow obstruction, sinusoidal dilatation, and hemosiderosis.

§ Mild portal, periportal, perisinusoidal, pericellular, or bridging fibrosis was frequently present in association with other diagnoses and was therefore not tabulated as an independent diagnosis.

¶ Includes granulomatous hepatitis, CMV-associated hepatitis, autoimmune liver disease/PBC, lobular hepatitis, and other inflammatory conditions.
