# Supplementary material for: Analytical and Clinical Validation of a Serum microRNA RT-qPCR Assay for Detection of Acute Cellular Rejection in Liver Transplant Recipients
Source: Diagnostics (Basel). 2026 Jul 9;16(14):2152. doi: 10.3390/diagnostics16142152 (PMC13409392; doi:10.3390/diagnostics16142152)
Supplement: Supplementary file 1 [file diagnostics-16-02152-s001.zip › HepatoTrack Analytical Clinical Validation Supplemental Material S5.pdf]

Supplemental File S5

Evaluation and Selection of Prediction Models for HepatoTrack™ Prediction Score Development

Several candidate prediction models were evaluated during model development to identify the most appropriate model for predicting biopsy-confirmed acute cellular rejection (ACR). Model development was performed in R using the caret and caretEnsemble packages. Sixteen candidate prediction models were evaluated, including Classification and Regression Trees (CART), logistic regression, linear regression, random forest, support vector machines, and other commonly used classification methods. Hyperparameter tuning was performed using 10-fold cross-validation.

Among the evaluated prediction models, logistic regression demonstrated diagnostic performance comparable to that of the final linear regression model, with identical classification accuracy in both the training cohort (72.3%) and the independent test cohort (81.1%). CART demonstrated the strongest performance among the evaluated machine-learning approaches and is presented here for comparison with the final linear regression model.

Supplemental File S5 Table S1. Performance of the CART Model in the Training Cohort

**True Positive (ACR)**	12
**False Positive**	7
**False Negative**	5
**True Negative (non-ACR)**	23
**Accuracy (95% CI)**	74.5% (59.7% - 86.1%)
**Sensitivity**	70.6%
**Specificity**	76.7%
**Pos Pred Value**	63.2%
**Neg Pred Value**	82.1%
**Balanced Accuracy**	73.6%

The prediction performance of the model on the independent test cohort (n=37) is as follows:

Supplemental File S5 Table S2. Performance of the CART Model in the Test Cohort

**True Positive (ACR)**	13
**False Positive**	5
**False Negative**	1
**True Negative (non-ACR)**	18
**Accuracy (95% CI)**	83.8% (68.0% - 93.8%)
**Sensitivity**	92.9%
**Specificity**	78.3%
**Pos Pred Value**	72.2%
**Neg Pred Value**	94.7%
**Balanced Accuracy**	85.6%

Final Model Selection

CART demonstrated predictive performance comparable to that of the final linear regression model. However, the linear regression model was selected for the HepatoTrack™ Prediction Score because it provided similar diagnostic performance while offering a simpler mathematical formulation and a readily interpretable continuous prediction score. Because the prediction model incorporated only two predictor variables, the potential performance gains achievable with more complex machine-learning approaches were expected to be limited. Given the modest sample size of the current study, a simpler linear regression model provided greater transparency and reproducibility while reducing the risk of fitting unstable relationships that may not generalize to future datasets. As larger prospective multicenter datasets become available, additional prediction models will be re-evaluated to determine whether they provide meaningful improvements in diagnostic performance.
