# Supplementary material for: Analytical and Clinical Validation of a Serum microRNA RT-qPCR Assay for Detection of Acute Cellular Rejection in Liver Transplant Recipients
Source: Diagnostics (Basel). 2026 Jul 9;16(14):2152. doi: 10.3390/diagnostics16142152 (PMC13409392; doi:10.3390/diagnostics16142152)
Supplement: Supplementary file 1 [file diagnostics-16-02152-s001.zip › HepatoTrack Analytical Clinical Validation Supplemental Material S6.pdf]

Supplemental File S6

Evaluation of Circulating miRNA Endogenous Controls in Liver Transplant Recipient Serum Samples

Circulating miRNA expression data were obtained from a previously published study [9]. In that study, miRNA profiling was performed on 318 serum samples collected from 69 liver transplant recipients enrolled in the Immune Tolerance Network immunosuppression withdrawal study (ITN030ST) and the Clinical Trials in Organ Transplantation study (CTOT-03). Expression of 752 human miRNA targets and six reference gene assays was measured using the miRCURY LNA Universal RT miRNA PCR platform (Exiqon, Vedbaek, Denmark).

Twenty candidate endogenous control miRNAs were selected from the literature and evaluated for expression stability (Supplemental File S6 Table S1).

Of the 318 serum samples analyzed, 257 passed quality-control criteria as described in the original publication. Among the 20 candidate endogenous controls evaluated, four miRNAs (miR-1260a, miR-361-5p, miR-23a-3p, and miR-221-3p) demonstrated coefficients of variation (CVs) below 4% and Ct ranges of less than 10 cycles. Among these four candidates, miR-23a-3p exhibited the highest expression level (lowest average Ct value), supporting its suitability as an endogenous normalizer.

Supplemental File S6 Table S1. Expression stability of candidate endogenous control miRNAs in liver transplant recipient serum samples

miRNA	Mean Ct Value	Ct Standard Deviation	Ct Range (Max - Min)	CV (%)
**hsa-miR-1260a**	33.2	1.0	6.2	2.9%
**hsa-miR-361-5p**	30.4	0.9	6.8	2.9%
**hsa-miR-23a-3p**	26.5	1.0	7.3	3.7%
**hsa-miR-221-3p**	28.7	1.1	7.4	3.9%
**hsa-miR-423-5p**	29.5	1.2	10.2	4.0%
**hsa-miR-320a**	29.8	1.2	10.6	4.2%
**hsa-miR-324-3p**	31.4	1.4	13.0	4.4%
**hsa-miR-186-5p**	29.5	1.3	12.6	4.5%
**hsa-miR-324-5p**	33.3	1.5	10.6	4.5%
**hsa-miR-191-5p**	30.2	1.4	13.4	4.6%
**hsa-miR-22-3p**	27.9	1.3	11.6	4.6%
**hsa-miR-26a-5p**	28.5	1.3	12.3	4.7%
**hsa-miR-484**	30.5	1.4	13.8	4.7%
**hsa-miR-181a-5p**	30.8	1.5	13.3	4.7%
**hsa-let-7a-5p**	29.1	1.4	15.0	4.9%
**hsa-miR-16-2-3p**	32.8	1.7	11.3	5.1%

**hsa-miR-93-5p**	27.6	1.5	12.9	5.4%
**hsa-miR-103a-3p**	27.7	1.6	15.4	5.6%
**hsa-miR-16-5p**	22.5	1.6	14.3	7.0%
**hsa-miR-451a**	21.7	1.8	14.8	8.3%

Additional analyses were performed to compare the expression of candidate endogenous control miRNAs between acute cellular rejection (ACR) and non-ACR samples (Supplemental File S6 Table S2). Although miR-23a showed a nominally significant difference between ACR and non-ACR groups (p = 0.0426, Wilcoxon rank-sum tests), this p-value was not adjusted for multiple comparisons. Furthermore, the magnitude of the observed difference (ΔCt = −0.4) was among the smallest observed across all candidate endogenous controls. Boxplots comparing expression levels between ACR and non-ACR groups for all 20 candidate endogenous controls are shown in Supplemental File S6 Figure S1.

Supplemental File S6 Table S2. Comparison of candidate endogenous control miRNA expression between ACR and non-ACR groups

	Average Ct Value (ACR Group)	Average Ct Value (non-ACR Group)	ΔCt (ACR – non-ACR)	*p* Value (ACR vs. non-ACR)
**hsa-miR-16.2-3p**	32.8	33.1	-0.3	0.30697
**hsa-miR-451a**	21.7	22.0	-0.3	0.2313
**hsa-miR-181a-5p**	30.7	31.1	-0.3	0.176437
**hsa-miR-103a-3p**	27.6	27.9	-0.3	0.118578
**hsa-miR-221-3p**	28.2	28.6	-0.4	0.098726
**hsa-miR-26a-5p**	28.4	28.7	-0.4	0.090582
**hsa-miR-191-5p**	29.7	30.2	-0.5	0.06037
**hsa-miR-23a-3p**	26.1	26.5	-0.4	0.04259
**hsa-miR-324-5p**	32.9	33.4	-0.5	0.037589
**hsa-miR-186-5p**	29.1	29.6	-0.5	0.023509
**hsa.let.7a-5p**	28.6	29.1	-0.5	0.013169
**hsa-miR-423-5p**	29.2	29.7	-0.5	0.009667
**hsa-miR-484**	29.9	30.5	-0.6	0.008568
**hsa-miR-16-5p**	22.0	22.6	-0.6	0.005959
**hsa-miR-324-3p**	31.0	31.6	-0.6	0.004363
**hsa-miR-93-5p**	27.2	27.8	-0.6	0.004022
**hsa-miR-361-5p**	29.8	30.4	-0.6	0.001677
**hsa-miR-22-3p**	27.4	28.2	-0.8	0.001549
**hsa-miR-320a**	28.9	29.8	-0.9	3.62E-05
**hsa-miR-1260a**	32.4	33.4	-1.0	2.17E-05

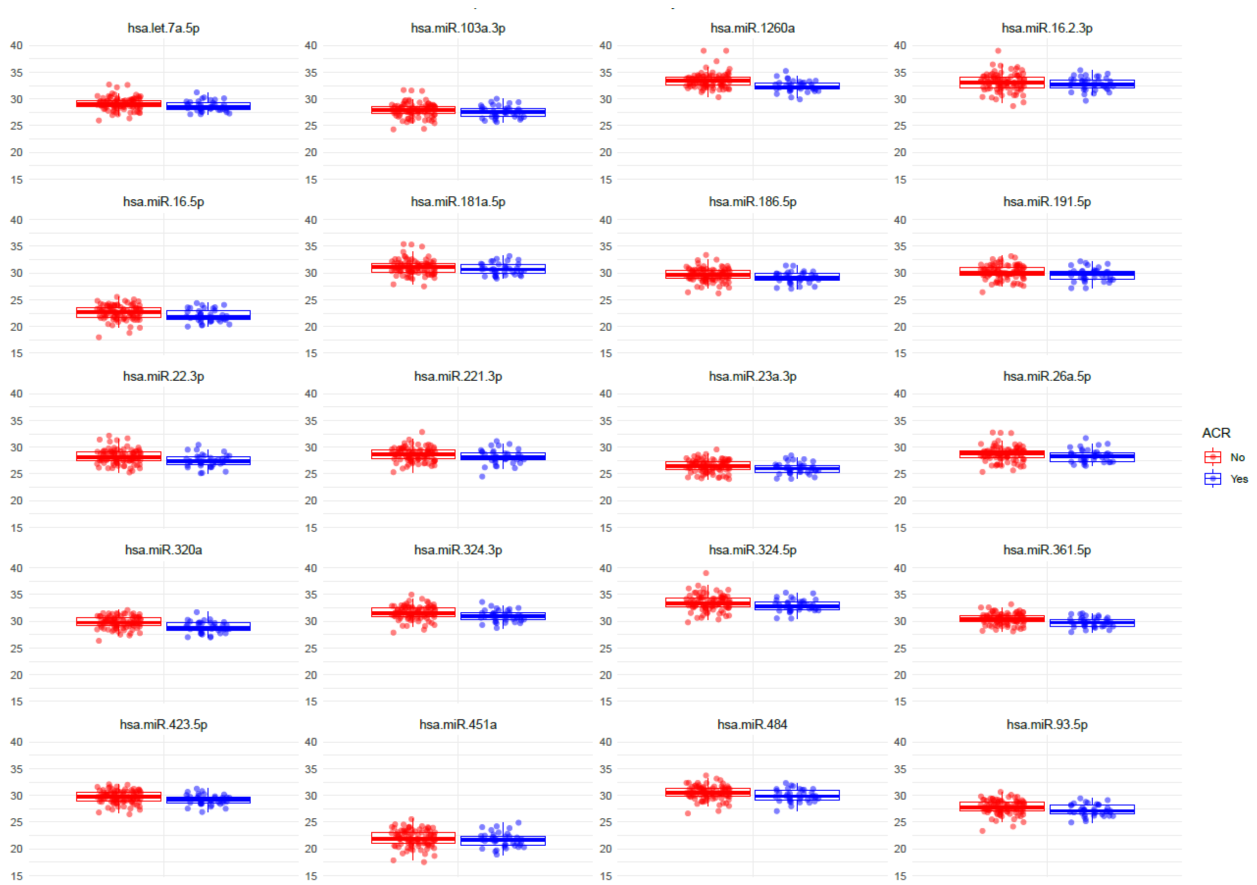

Supplemental File S6 Figure S1. Boxplots of candidate endogenous control miRNA expression in ACR and non-ACR serum samples. Expression levels are shown for the 20 candidate endogenous control miRNAs evaluated in this study. Y-axis: Ct value. X-axis: ACR status.
